# Mapping of lymph node dissection determined by the epicenter location and tumor extension for esophagogastric junction carcinoma

**DOI:** 10.3389/fonc.2022.913960

**Published:** 2022-11-28

**Authors:** Rong Liang, Xiaogang Bi, Daguang Fan, Qiao Du, Rong Wang, Baoyu Zhao

**Affiliations:** ^1^ Department of Digestive System, Shanxi Provincial People’s Hospital, Taiyuan, China; ^2^ Chinese Research Group of Esophagogastric Junction Carcinoma, Department of General Surgery, Shanxi Provincial People’s Hospital, Taiyuan, China

**Keywords:** esophagogastric junction carcinoma, epicenter location, tumor extension, lymph node metastasis, lymph node dissection, therapeutic efficacy

## Abstract

**Backgrounds:**

Previous studies identified the extent of lymph node dissection for esophagogastric junction (EGJ) carcinoma based on the metastatic incidence. The study aimed to determine the optimal extent and priority of lymphadenectomy based on the therapeutic efficacy from each station.

**Methods:**

The studies on the lymph node metastasis (LNM) and therapeutic efficacy index (EI) for EGJ carcinomas were identified until April 2022. The obligatory stations with the LNM rates over 5% and therapeutic EI exceeding 2% should be routinely resected for D2 dissection, whereas the optional stations with EI between 0.5% and 2% should be resected for D3 dissection in selective cases.

**Results:**

The survey yielded 16 eligible articles including 6,350 patients with EGJ carcinoma. The metastatic rates exceeded 5% at no. 1, 2, 3, 7, 9, 11p, and 110 stations and were less than 5% in abdominal no. 4sa~6, 8a, 10, 11d, 12a, and 16a2/b1 and mediastinal no. 105~112 stations. Consequently, obligatory stations with EI over 2% were largely determined by the epicenter location and located at the upper perigastric, lower mediastinal, and suprapancreatic zones, corresponding to those with rates of LNM over 5%. Consistent with the LNM rates less than 5%, the optional stations with EI between 0.5% and 2% were largely dependent on the degree of tumor extension toward the lower perigastric, splenic hilar (grecurvature), para-aortic (less curvature of the cardia), and middle or upper mediastinal zones.

**Conclusions:**

The obligatory stations can be resected as an “envelope-like” wrap by transhiatal proximal gastrectomy with lower esophagectomy, whereas the optional stations for dissection are indicated by the tumor extension. The extended gastrectomy is required for the lower perigastric in the stomach-predominant tumor with gastric involvement exceeding 5.0 cm, para-aortic dissection in the less curvature-predominant tumor and splenic hilar dissection in the grecurvature-predominant tumor whereas transthoracic subtotal esophagectomy is required for complete mediastinal dissection and adequate negative margin in the esophagus-predominant tumor with esophageal invasion exceeding 3.0 cm.

## Introduction

**Figure 1:**
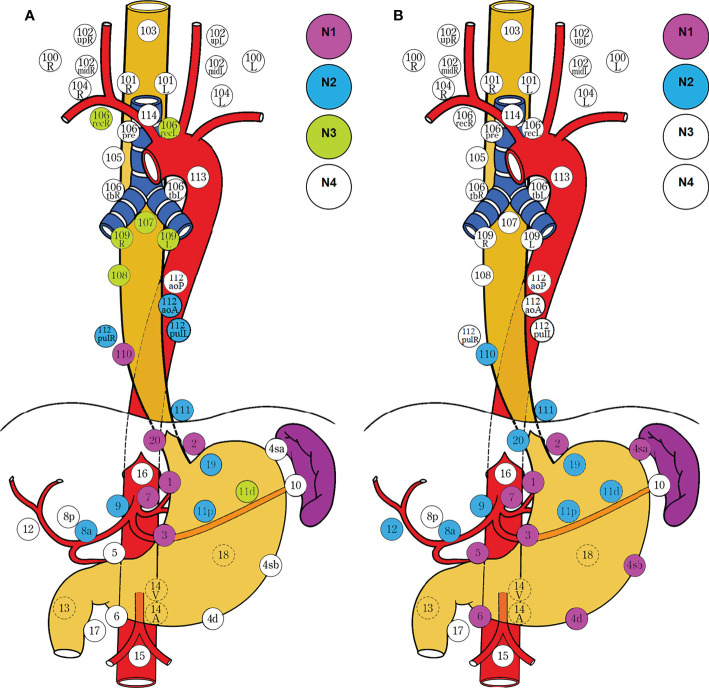
Lymph node dissection for EGJ cancer determined by the surgical approach. Two-field D3 dissection *via* transthoracic esophagectomy was adopted by the Japanese Classification of Esophageal Cancer (11^th^ edition) **(A)**, and D2 dissection *via* transhiatal extended gastrectomy was employed by the Japanese Gastric Cancer Treatment Guidelines (5^th^ edition) **(B)**. The former ensures adequate proximal margin and complete mediastinal dissection, whereas the latter ensures extensive abdominal dissection and lower morbidity. The survivals are comparable between the two different procedures for dissection. N1, metastasis involving only group 1 lymph nodes; N2, metastasis to group 2 nodes, regardless of involvement of group 1 nodes; N3, metastasis to group 3 nodes, regardless of involvement of group 1 or 2 nodes; N4, metastasis to distant (group 4) nodes, regardless of whether any other group(s) of regional lymph nodes are involved or not. D1, complete dissection of N1 nodes, but no or incomplete dissection of N2 nodes. D2, complete dissection of N1 and N2 nodes, but no or incomplete dissection of N3 nodes. D3, complete dissection of N1, N2, and N3 nodes. The stations for dissection are marked with colored circles according to the grading of lymph node metastasis. The left side (L) and the right side (R) should be distinguished for 101, 102, 104, 106rec, 106tb, 109, and 112pul.

In recent years, the incidence of esophagogastric junction (EGJ) adenocarcinoma has increased gradually worldwide (1–5). Siewert’s classification defines EGJ cancer as adenocarcinoma with an epicenter within 5.0 cm of the EGJ, as follows (6): type I, 5~1 cm above the EGJ; type II, 1 cm above to 2 cm below the EGJ; and type III, 2~5 cm below the EGJ. In Japan, Nishi’s classification (7) defines EGJ cancer as the epicenter located within 2.0 cm of the EGJ, which is almost the same as Siewert type II carcinoma. A worldwide consensus regarding the surgical procedure has been reached for Siewert I and III type carcinoma from two randomized controlled trials (8–11). Ivor Lewis esophagectomy with complete mediastinal dissection is the standard treatment for Siewert I carcinoma (10, 11), transhiatal extended gastrectomy with excessive perigastric dissection for Siewert III carcinoma (8, 9). However, Siewert II carcinoma has been treated as esophageal carcinoma or gastric cancer depending on the surgeon’s preference (12, 13). Even for Siewert II tumors with the same location, thoracic surgeons prefer transthoracic esophagectomy, whereas gastric surgeons prefer transabdominal extended gastrectomy (13). The former ensures adequate proximal resection margin and complete mediastinal dissection with a great impact on the survival (**Figure 1A**), whereas the latter ensures lower morbidity and extensive abdominal lymph node dissection based on the main lymphatic pathways from the cardia downward to the para-celiac nodes (**Figure 1B**). Indisputably, whether the dissection in a certain area is worthwhile remains dependent on the metastatic incidence and therapeutic efficacy index of such procedures in terms of benefit–risk balance (12). The fact is that the long-term survival rates between the two procedures are comparable from the two randomized controlled trials (8–11). However, the extent of lymph node dissection is obviously different between transthoracic esophagectomy and transhiatal extended total gastrectomy (12) (**Figure 1A vs. 1B**). Finally, the extent of lymphadenectomy for EGJ cancer has been poorly defined according to the metastatic rates from multiple retrospective studies (14, 15). They could not accurately determine the survival benefit from the dissection of each metastatic station due to selection bias and sample size.

This survey aimed to identify the optimal extent and priority of lymph node dissection for EGJ cancer, based on the incidence of lymph node metastasis (LNM) and therapeutic efficiency index (EI) of estimated survival benefit from nodal dissection of each station by reviewing findings from the latest studies. Additionally, EGJ cancer defined by Nishi’s classification corresponds to Siewert II cancer according to the Japanese Classification of Esophageal Cancer and Japanese Classification of Gastric Carcinoma (14, 15). Therefore, EGJ cancer according to Nishi’s definition was assigned into the same category as Siewert II cancer in the data analysis.

## Materials and methods

### Definition and classification of EGJ cancer

**Figure 2:**
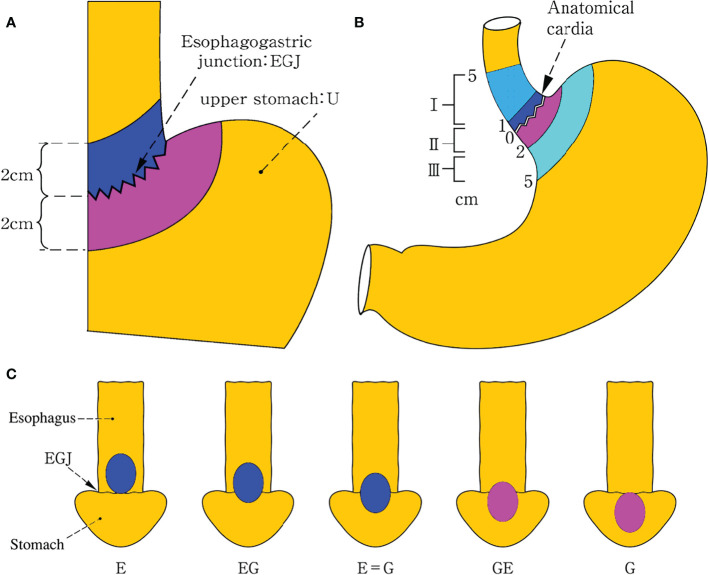
Definition and description of EGJ cancer. Nishi’s classification **(A)** adopted by the TNM classification of malignant tumor 8th edition, which defined EGJ cancer as adenocarcinoma with an epicenter located within 2.0 cm above and below from the EGJ and corresponded to Siewert type II cancer defined by Siewert’s classification **(B)**. The terms “E, EG, E=G, GE, and G” were used to describe the subtypes depending on the epicenter location at the oral “E” and anal “G” portions of the EGJ. E, EG, and E=G were scheduled as the esophagus-predominant tumor (E≥G) and G and GE as the stomach-predominant tumor (G>E) among five types **(C)**. Both Siewert’s and Nishi’s classification clearly settled the epicenter location within 2 cm above and below the EGJ, irrespective of tumor size in the former and histological type in the latter as well as tumor extension in both. EGJ esophagogastric junction.

Nishi’s classification was adopted by the TNM classification of malignant tumor 8th edition (16); EGJ cancer was defined as adenocarcinoma with an epicenter located between 2.0 cm proximal to and distal from the EGJ and corresponded to Siewert type II cancer defined by Siewert’s classification (7) (**Figures 2A, B**). The terms “E, EG, E=G, GE, and G” were used to describe the subtypes depending on the epicenter location at the oral E and anal G portions of the EGJ. Both of the epicenter location and tumor extension effect on the nodal metastasis should be taken into consideration for the optimal dissection. E, EG, and E=G were scheduled as the esophagus-predominant tumor (E≥G) (15) and G and GE as the stomach-predominant tumor (G>E) (14) (**Figure 2C**).

### Literature search strategy

A systematic literature search was undertaken using Medline (*via* PubMed) and the Cochrane library databases up to December 2019. The search terms “esophagogastric junction”, “gastroesophageal junction”, “Siewert classification”, “Nishi’s classification”, “cardiac carcinoma”, “carcinoma”, “lymph node metastasis”, “lymph node dissection”, and Medical Subject Headings were used in combination with the Boolean operators “AND” or “OR”.

### Study selection

**Figure 3:**
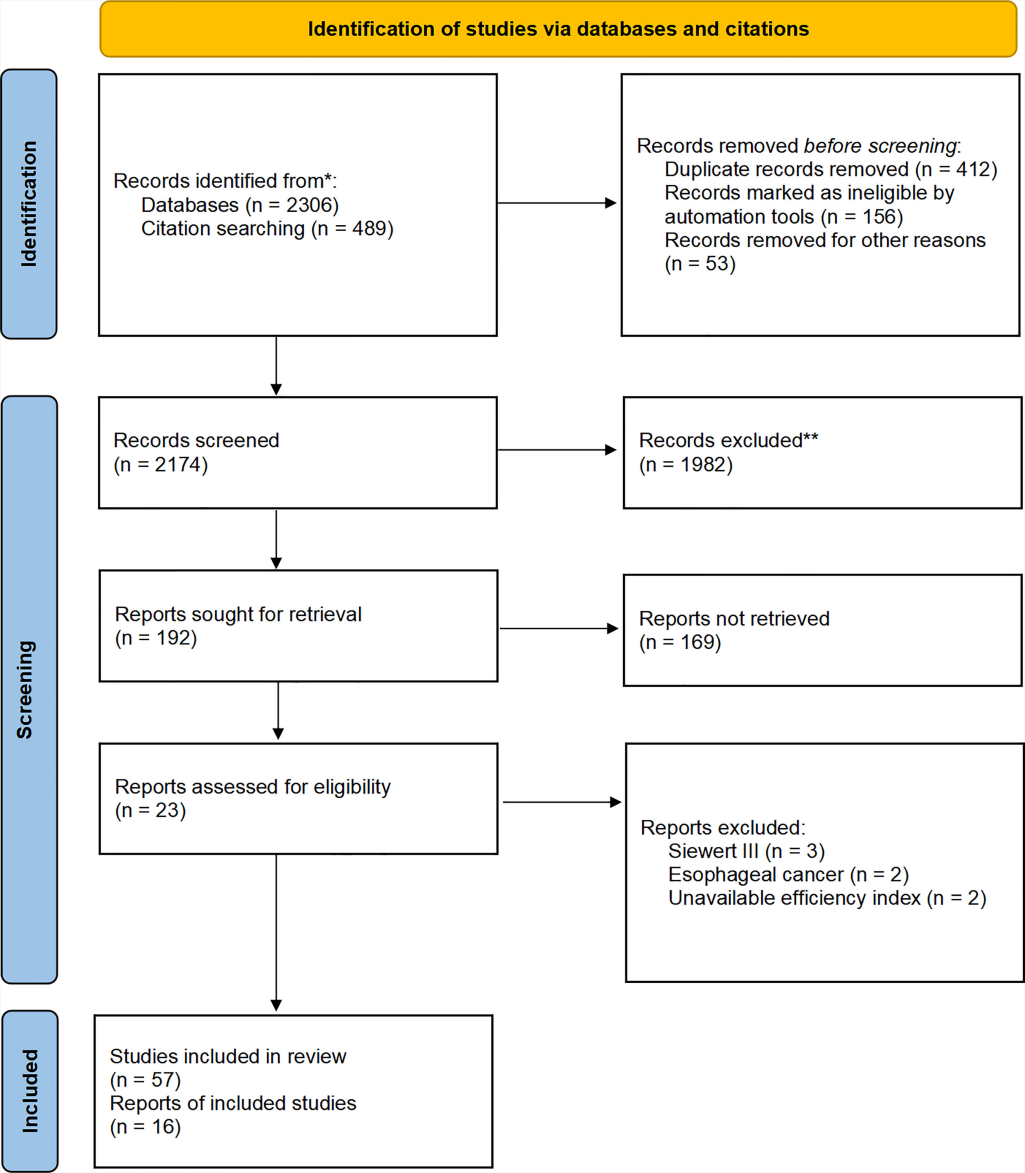
Flow diagram of included studies. *From:* Page MJ, McKenzie JE, Bossuyt PM, Boutron I, Hoffmann TC, Mulrow CD, et al. The PRISMA 2020 statement: an updated guideline for reporting systematic reviews. BMJ 2021;372:n71. doi: 10.1136/bmj.n71.

After eliminating duplicates, titles and abstracts were carefully screened by two of the authors (Baoyu Zhao and Rong Liang) to determine their suitability for inclusion in the pooled analysis. The full text of relevant articles was independently retrieved and assessed for inclusion (**Figure 3**). Primary articles with EGJ cancer patients undergoing esophagectomy or gastrectomy or esophagogastrectomy plus lymph node dissection were eligible for inclusion. Just the studies evaluating the metastatic incidence and the therapeutic value index of estimated benefit from each nodal station were included. Esophagus cancer, gastric carcinoma, Siewert I or III carcinoma, case reports, studies with fewer than 30 patients, reviews, posters, letters, comments, abstracts, surgical techniques, studies published before 2022, and studies in a language other than English were excluded. In addition, studies without nodal station and 5-year survival rate of metastatic station were excluded. Additionally, some references from these articles were also retrieved and added, to become more comprehensive. Any discordance regarding inclusion between the two authors was resolved by consensus.

### Data extraction and presentation

Two independent reviewers (Rong Liang and Baoyu Zhao) extracted data and reached an agreement by discussion with conflict resolution by a third reviewer (Rong Wang). Major extracted items included study design, first author, publication year, sample size, histology, classification, lymph node metastasis from each station, and therapeutic efficacy index from each station. The extracted data were presented per study. The grouping of mediastinal and abdominal lymph node stations adopted the Japanese classification of esophageal carcinoma (11th edition) (15) and Japanese classification of gastric cancer (3th edition) (17), respectively (**Table 1** **)**.

**Table 1:** The stations of regional lymph nodes from EGJ cancer.

**(1) Cervical LNs**		No. 1	Right paracardial LNs
No. 100	Superficial LNs of the neck	No. 2	Left paracardial LNs
No. 101	Cervical paraesophageal LNs	No. 3a	Lesser curvature LNs along the left gastric artery
No. 102	Deep cervical LNs	No. 3b	Lesser curvature LNs along distal part of right gastric artery
No. 103	Peripharyngeal LNs	No. 4sa	LNs along the short gastric vessels
No. 104	Supraclavicular LNs	No. 4sb	LNs along the left gastroepiploic artery
**(2) Thoracic LNs**		No. 4d	LNs along the right gastroepiploic artery
No. 105	Upper paraesophageal LNs	No. 5	Suprapyloric LNs
No. 106	Thoracic paratracheal LNs	No. 6	Infrapyloric LNs
No. 106rec	Recurrent nerve LNs	No. 7	LNs along the left gastric artery
No. 106recL	Left recurrent nerve LNs	No. 8a	LNs along the common hepatic artery (anterior group)
No. 106recR	Right recurrent nerve LNs	No. 8p	LNs along the common hepatic artery (posterior group)
No. 106pre	Pretracheal LNs	No. 9	LNs along the celiac artery
No. 106tb	Tracheobronchial LNs	No. 10	LNs at the splenic hilum
No. 106tbL	Left tracheobronchial LNs	No. 11	LNs along the splenic artery
No. 106tbR	Right tracheobronchial LNs	No. 11p	LNs along the proximal splenic artery
No. 107	Subcarinal LNs	No. 11d	LNs along the distal splenic artery
No. 108	Middle paraesophageal LNs	No. 12	LNs in the hepatoduodenal ligament
No. 109	Main bronchus LNs	No. 12a	LNs along the proper hepatic artery
No. 109L	Left main bronchus LNs	No. 12b	LNs along the bile duct
No. 109R	Right main bronchus LNs	No. 12p	LNs along the portal vein
No. 110	Lower paraesophageal LNs	No. 13	LNs on the posterior surface of the pancreatic head cranial to the duodenal papilla
No. 111	Supradiaphragmatic LNs	No. 14	LNs along the superior mesenteric vessels
No. 112	Posterior mediastinal LNs	No. 15	LNs along the middle colic vessels
No. 112aoA	Anterior thoracic paraaortic LNs	No. 16a1	LNs in the aortic hiatus
No. 112aoP	Posterior thoracic paraaortic LNs	No. 16a2	LNs around the aorta (from the upper margin of the celiac trunk to the lower margin of the left renal vein)
No. 112pul	Pulmonary ligament LNs	No. 16b1	LNs around the aorta (from the lower margin of the left renal vein to the upper margin of the inferior mesenteric artery)
No. 113	Ligamentum arteriosum LNs	No. 16b2	LNs around the aorta (from the upper margin of the inferior mesenteric artery to the aortic bifurcation)
No. 114	Anterior mediastinal LNs	No. 19	Infradiaphragmatic LNs along the subphrenic artery
**(3) Abdominal LNs**		No. 20	LNs in the esophageal hiatus of the diaphragm

The lymph node station adopted the Japanese Classification of Esophageal Carcinoma (11th edition) and Gastric Cancer (3th edition). LNs, lymph nodes. The left side (L) and the right side (R) should be distinguished for nos. 101, 102, 104, 106rec, 106tb, 109, and 112pul.

### Therapeutic efficacy of lymph node dissection and statistical analysis

To evaluate the therapeutic efficiency of nodal dissection at each station, we adopted the efficacy index (EI) calculated by multiplying the incidence of lymph node metastasis (LNM) to each station by the corresponding 5-year overall survival rate in the patients with metastasis at that station (18, 19). Thus, the survival benefit was evaluated without any concept of staging of lymph node metastasis.


$$LNM(\%)=\left(\frac{Number\;of\;patients\;with\;metastasis\;at\;each\;station}{Number\;of\;total\;dis\sec ted\;patients\;at\;that\;station\;}\right)\times 100\%$$

$$EI=\left(\frac{Number\;of\;patients\;with\;\;metastasis\;at\;each\;station}{Number\;of\;total\;dis\sec ted\;patients\;at\;that\;station}\right)\times \left(\begin{array}{l} the\;5-year\;overall\;survival\;rates\;for\\ patients\;with\;metastasis\;at\;that\;station \end{array}\right)$$

Medians with interquartile ranges (IQRs) expressed the values of LNM and EI at each station. The nodal stations were divided into four categories according to the EI after dissection (18): EI-1 category (should be resected in every case), EI exceeding 5%; EI-2 category (should be resected as far as practicable), EI between 2% and 5%; EI-3 category (need not be resected if a patient is at high risk for mortality and morbidity), EI from 0.5% to 2%, divided into EI-3A (1%~2%) and EI-3B (0.5%~1%); and EI-4 category (need not be resected in any case), EI less than 0.5%. Based on the incidence of LNM (lymph node ratio), the stations were grouped into three categories for dissection (18–20): LNM-1 category (strongly recommended), rate exceeding 10%; LNM-2 category (weakly recommended), rate from 5% to 10%; and LNM-3 category (not recommended), rate less than 5%. The cutoff values of LNM rates were determined by the grouping of the regional lymph nodes based on the efficacy index of estimated survival benefit in the 13th Ed. Japanese Classification of Gastric Carcinoma (15, 19, 20). The lymph nodes were strictly classified into the category of D grading by the efficacy index value from the retrospective study, but they can be classified by the metastatic rates from the prospective study unless the index value was provided. According to the Japanese Classification of Esophageal Cancer and Japanese Gastric Carcinoma Treatment Guidelines, both LNM-1, 2 and EI-1, 2 nodes were scheduled as the obligatory categories of stations for D2 dissection; LNM-3 and EI-3 nodes as the optional categories for D3 dissection (**Table 2**).

**Table 2:** The grading of lymph node dissection according to metastatic rate and efficiency index.

**Category**	**Obligatory stations**		**Optional stations**		**Distant metastasis**
**LNM**	**LNM-1**	**LNM-2**	**LNM-3**		**LNM-3**
Cutoff (%)	≥10%	5%~10%	<5%		
D grading	Strongly recommended for D1	Weakly recommended for D2	Not recommended for D3		Not recommended for D4
**EI**	**EI-1**	**EI-2**	**EI-3**		**EI-4**
Cutoff (%)	>5%	2%~5%	**E-3A:**1%~2%	**E3-B:** 0.5%~1%	<0.5%
D grading	Frequent metastasis Good prognosis after dissection Should be resected in any case	Intermediate metastasis and prognosis after dissection Resected as far as practicable	Rare metastasis Poor prognosis after dissection Need not be resected at high risk		Distant metastasis Need not be resected in any case

LNM, lymph node metastasis; EI, efficiency index = LNM (%) × 5-year overall survival rate (%)/100; D, dissection; D1, complete dissection of obligatory EI-1 nodes only; D2, complete dissection of obligatory EI-1 and EI-2 nodes, but no or incomplete dissection of optional EI-3 nodes; D3, complete dissection of EI-1, EI-2, and EI-3 nodes; D4, complete dissection of EI-1, EI-2, EI-3 and EI-4 nodes.

## Results

### Characteristics of the included study on EGJ carcinoma

In the qualitative synthesis, a systematic search yielded 16 eligible and retrospective studies including 6,350 patients with EGJ carcinoma according to Siewert or Nishi’s classification. The majority of patients from included studies have advanced adenocarcinoma (pT2~T4 stages) with an epicenter located at 2.0 cm above and below the EGJ. In addition, some studies were of greater interest in the dissection at the upper perigastric, mediastinal, lower perigastric, para-aortic and splenic hilar stations. Fewer included studies performed the subgroup analysis by tumor size, tumor stage, and tumor extension. The values of LNM and EI are listed in **Table 3**. The data from the studies only with Siewert I and III type tumors were excluded, because they were assigned into Barrett’s esophageal adenocarcinoma and sub-cardia gastric adenocarcinoma, respectively, and there has been consensus on the lymph node dissection.

**Table 3:** Metastatic rate and therapeutic efficiency index of each nodal station for esophagogastric junction carcinoma.

Study		Year	Classification		Sample	No. 1		No. 2		N0. 3		No.4sa		No.4sb		No.4d		No. 5		No. 6		No. 7		No.8a		No. 9		No. 10		No.11p	
						LNM	EI	LNM	EI	LNM	EI	LNM	EI	LNM	EI	LNM	EI	LNM	EI	LNM	EI	LNM	EI	LNM	EI	LNM	EI	LNM	EI	LNM	EI
H.Yamashita(21)		2011	II	AC	225	38.2	13.8	23.1	7	35.1	13.7	4	1	1.3	0	0	0	0.6	0	1.2	0	20.9	3.8	6.2	2.2	10.2	1.5	4.1	0.7	11.1	2.6
S. Mine(22)		2012	II	AC	150	44.7	18.8	34.7	15.3	46	20.7	3.4	0					1	0	0	0	28	14.8	2.3	0	12.7	1.4	4.7	1.9	16.5	3.4
M.Yura(23)		2018	Nishi	AC/SCC	84/26	27.3	14.3	20.9	7.7	23.6	10	1.3	1.3	0	0	0	0	0	0	0	0	16.4	6.4	2.7	0	5.5	2.8	8.3	4.2	3.3	0
Peng J(24)		2015	II	AC	192	60	18	60	18	58.1	23.3	30.6	5.8					15.4	0	12.5	0	48	12	12.8	4.2	27	15.4	2.7	2.7	20	6.6
K. Fujitani(25)		2012	II	AC	86	61.6	16.3	31.4	5.8	52.3	11.6	7	3.5	3.5	1.2			1.2	0	3.5	1.2	25.9	5.9	8.5	2.4	8.8	1.3	2.9	1.5	20.5	8.2
H. Goto(26)		2012	II	AC	42	59.5	29.9	19	8.9	57.1	30.3	2.4	0	4.8	2.4	0	0	2.4	2.4	2.4	0	30.9	18.1	2.4	0	23.8	7.1	4.8	0	16.7	5.6
T. Matsuda(27)		2014	Nishi	AC/SCC	53/15	29.4	10.3	17.6	1.4	23.5	10.3	3.6	3.6									16.1	4.3								
H. Yabusaki(28)		2013	II	SCC	51	40.8	11.3	18.4	6.1	34.7	11.6	0	0	0	0	0	0	0	0	2.8	0	19.1	6.4	2.3	0	8.9	0	0	0	12.8	2.6
			II	AC	72	45.8	11.1	22.2	8.3	40.3	11.1	1.8	0	1.8	0	3	1.5	1.7	0	4.8	1.6	28.2	5.6	7.6	1.5	15.9	1.4	7	2.3	9.4	1.6
Jia-Bin Wang(29)		2017	II	AC	385	24.9	11.6	29.5	13.5	55	28.7	8.8	2.4					3.5	0.5	12.5	0.3	36.7	17.3	8.3	2.3	20.4	8.5	8.9	1.8	11	4.7
Hui-Hua Cao(30)		2018	II	AC	141													1.4	0	0.7	0										
S. Hasegawa(31)		2013	II	AC	95	42.1	19.5	26.3	9.1	45.3	21.5	1.1	0	2.3	0	3.5	0	0	0	0	0	32.6	10.9	6.8	2.8	3.6	0	4.7	1.6	17.7	3.3
H. Goto(32)		2015	II	AC	92	39.1	14.3	13	2.1	37	16.8	2.2	0	2.2	1.1	0	0	2.7	0	0	0	21.7	8.8	3.5	1.8	17.2	3.9	5.8	0	13.2	2.5
H. Yamashita(33)		2016	Nishi	SCC/AC	2807																										
			EG	AC	397	5	3.5	2	1.4	7.1	5	0.3		0.3		0	0	0	0	0	0	3.8	2.8	0.5	0.5	1	1.4	0.3	0	0.3	0
			EG	AC	237	34.6	9.2	16.5	5.7	28.7	13.4	1.3		1.3		0.8		0	0	0	0	17.7	4.8	3.8	0.9	6.8	3.9	0.8	0	4.2	3.3
			EG	SCC	150	6.7	6	4	4.3	10.7	10.5	0	0	0	0	0	0	0	0	0	0	6	5.1	0.7		0.7	0	0	0	0	0
			EG	SCC	177	29.9	14	19.2	12.5	24.3	13.4	0.6		0	0	0	0	0	0	0	0	21.5	10.6	2.8	2.8	5.6	6.4	0	0	2.3	2.4
			GE	AC	1018	4	3	1.6	1.6	3.9	3.1	0.1		0	0	0.2	0.4	0	0	0.1	0	1.1	1	0.2		0.9	0.6	0.1	0.9	0.5	0.6
			GE	AC	775	30.5	13.4	15	7.2	29.5	15.2	0.8	0.5	0.5		0.4	0.4	0.5	0	0.9	0.6	12.5	4.4	3	1.1	3.5	0.8	0.9	1.1	4.5	2.8
T. Yoshikawa(34)		2014	II	AC	381	39.8	16.2	30.8	13.6	41.5	19.8	4.3	1	2.7	0.3	2.9	1.1	1.7	0	0.8	0.4	26.7	11.7	4.9	0.7	11.7	1.7	9.5	1.8	17.2	4.7
			II	SCC	50	44.7	17.1	25	7.4	34.7	7.2	0	0	0	0	16.7		0	0	0	0	17.4	4.4	2.4	0	7.1	2.4	0	0	2.9	0
H. Fujita(19)		2007	Nishi	AC/SCC	1289																										
			EG	AC/SCC	130/393	33.7	13.2	23.3	7.6	22.2	8.1	2.1	0.5	1.5	0.9	1.5	0.8	1.5	0.8	1.1	1	18.9	6.8	6.7	2.2	5.7	2	0.4	0.2	5.5	2.6
			GE	AC/SCC	694/72	30	14	19.8	9.3	24.9	10	6	3	3.7	2.8	2.6	1.5	3	1.8	2.3	1.6	17.1	5.5	6.5	3.3	6.8	2.7	3.9	1.3	7.7	2.9
Ming-Zhi Cai(35)		2019	II	AC	167	35.3	13.2	30.5	9	55.1	22.8	2.8	0	1.2	0.6	3	0	0.6	0	0	0	19.8	7.8	6.6	1.2	11.4	3.6	5	0	10.8	3.2
Study		Year	Classification		Sample	No.11d		No.12a		16a2/b1		No. 19		No. 20		No. 110		No. 111		No. 112		No. 107		No. 108		No. 109		No. 105		No. 106	
						LNM	EI	LNM	EI	LNM	EI	LNM	EI	LNM	EI	LNM	EI	LNM	EI	LNM	EI	LNM	EI	LNM	EI	LNM	EI	LNM	EI	LNM	EI
H.Yamashita(21)		2011	II	AC	225	6.9	2.2	0	0	11	1.4					7.4	1.8	0	0	0	0										
S. Mine(22)		2012	II	AC	150			8	0	17	3.2					18	6.3														
M.Yura(23)		2018	Nishi	AC/SCC	84/26	0	0	0	0							19.6	12.2	8.2	2.1	8.2	2.1	11.4	0					16.7	8.9	16.7	8.9
Peng J(24)		2015	II	AC	192			8.3	0							17.2	5.3	13.3	0	3	3	6.9	1.7	17	9.7	2.7	2.7				
K. Fujitani(25)		2012	II	AC	86																										
H. Goto(26)		2012	II	AC	42	4.8	0	0	0			9.5	4.8	4.8	2.4																
T. Matsuda(27)		2014	Nishi	AC/SCC	53/15											18.2	4.5														
H. Yabusaki(28)		2013	II	SCC	51	0	0	16.7	0	28.5	0					30.4	4.5	8.3	0	8.3	0	4.8	2.4	4.8	2.4						
			II	AC	72	0	0	0	0	25	5					11.7	2.9	0	0	12.5	0	0	0	0	0						
Jia-Bin Wang(29)		2017	II	AC	385			2.4	0																						
Hui-Hua Cao(30)		2018	II	AC	141																										
S. Hasegawa(31)		2013	II	AC	95	2.6	1.3	3.3	0	5.4	0					12.8	7.7	2.3	0	0	0			15.4	0						
H. Goto(32)		2015	II	AC	92	3.1	0	0	0			17.4	0	9.5	4.8																
H. Yamashita(33)		2016	Nishi	SCC/AC	2807																										
			EG	AC	397	0.3	0			0	0	0.3	0	0.8	0.8	0.5	1.1	0.3	0	0.5	1.1	0		0.8	2.5	0		0.3		0.3	0
			EG	AC	237	2.1	2.4			0.8		0	0	0	0	5.1	1.9	1.7	1.2	1.3	0	0.4	2.6	1.3	0	1.7	0	0.4		0	
			EG	SCC	150	0	0			0	0	0	0	0.7	0	2.7	3.3	0.7	1.1	0		0		1.3		0		0		5	2.2
			EG	SCC	177	0	0			0	0	0	0	1.7		11.9	7.8	3.4	0	2.3	1.1	1.7	1.2	4	2	2.8	3.8	1.1		5.1	0.7
			GE	AC	1018	0	0			0	0	0	0	0	0	0		0		0		0		0		0		0		0	
			GE	AC	775	1.3	1.7			0.6	0	0.8	3.1	0.4	0	0.9	0	0.5	0	0.3		0		0.3	0	0		0		0.1	0
T. Yoshikawa(34)		2014	II	AC	381	6.3	1.7	1.4	0	14.4	2.4	4.9	0	1.5	0	18.1	6					20	5					15.8			
			II	SCC	50	0	0	25		11.1		6.3		0	0	25	4.2					19	0					8.3	0		
H. Fujita(19)		2007	Nishi	AC/SCC	1289																										
			EG	AC/SCC	130/393			0.2	0	1	0.4	0.6	0.4	1.3	0	14.3	5	6.7	3.7	5.4	1.6	3.8	1	6.9	2.4	5.2	1.5	2.1	0	9.8	1.8
			GE	AC/SCC	694/72			0.7	0.3	2.5	0.7	0.5	0.4	1	0.7	5.6	1.1	3.1	0.6	1.2	0.7	0	0	1.4	0.6	0	0	0.1	0	0.8	0.4
Ming-Zhi Cai(35)		2019	II	AC	167	2.4	0	1.2	0			8.6	0	6.2	1.6																

LNM lymph node metastasis, EI efficiency index=LNM (percentage) × 5-year overall survival rate (percentage)/100, AC adenocarcinoma, SCC squamous cell carcinoma.

### Lymph node metastasis and therapeutic efficacy index

**Figure 4:**
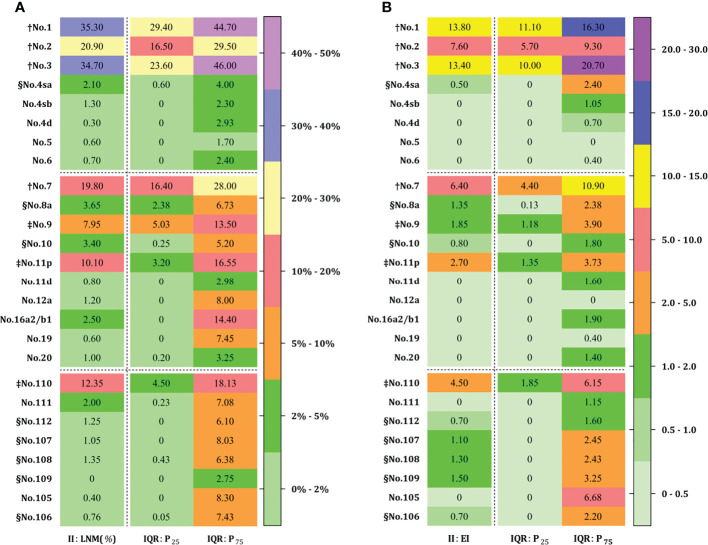
Lymph node metastasis **(A)** and efficiency index **(B)** for advanced EGJ carcinomas at each station. † LNM-1 ≥10% EI-1 ≥5; ‡ LNM-2 ≥5% EI-2 ≥2; § LNM-3<5% EI-3 ≥0.5 (EI-3B ≥1.0 and EI-3B ≥0.5). LNM-1 and LNM-2 nodes with LNM ≥10% and 5% are designated as obligatory stations for dissection in any case **(A)**, and corresponding to EI-1 and EI-2 nodes with EI ≥5 and 2 **(B)**, while both EI-3A and EI-3B nodes with EI ≥1 and EI ≥0.5 are designated as optional stations. The obligatory stations for dissection is determined by the epicenter location, whereas the optional stations remain dependent on the tumor extension. The obligatory nodes are located at the no. 1, 2, 3, 7, 9, 11p, 110 stations, whereas optional nodes are located in abdominal no. 4sa~6, 8a, 10, 11d, 12a, and 16a2/b1 stations and mediastinal no. 105~112 stations. Accordingly, EI-1 and EI-2 nodes were consistent with those with a rate over 5%. In contrast, optional nodes with a rate of less than 5% were assigned to EI-4 less than 0.5% and EI-3 less than 2%, respectively. EGJ, esophagogastric junction; II, Siewert type II; LNM, lymph node metastasis; EI, efficiency index; IQR, interquartile range.

The rate of lymph node metastasis and the efficacy index were expressed as LNM and EI (median, IQR: P_25_~P_75_), respectively, from each station as shown in **Figure 4**. The perigastric zones with median LNM rates exceeding 10% were assigned to LNM-1 nodes including no. 1 (35.3%, 29.4%~44.7%), no. 2 (20.9%, 16.5%~29.5%), no. 3 (34.7%, 23.6%~46.0%), no. 7 (19.8%, 16.4%~28.0%), and no. 11p (10.1%, 3.2%~16.6%) stations and LNM-2 nodes exceeding 5% included no. 9 (8.0%, 2.4%~6.7%) (**Figure 4A**). LNM-3 nodes with rates of less 5% included No. 4sa~6 (0.7~2.1%, 0%~4%), no. 8a (3.7%, 2.4%~6.7%), no. 10 (3.4%, 0.3%~5.2%), no. 11d (0.8%, 0%~3%), no. 12a (1.2%, 0%~8%), no. 16a2/b1 (2.5%, 0%~14.4%), no. 19 (0.6%, 0%~7.5%), and no. 20 (1.0%, 0.2%~3.3%) stations. Obligatory EI-1 and EI-2 nodes with EI exceeding 2% were located at the no. 1 (13.8), no. 2 (7.6), no. 3 (13.4), no. 7 (6.4), no. 9 (1.9), and no. 11p (2.9) stations (**Figure 4B**), the order being consistent with that of the frequency of nodal metastasis. Optional EI-3 nodes included no. 4sa (0.5), no. 8a (1.4), and no. 10 (0.8) stations, whereas the rest of the distal perigastric No. 4sb~6 and No. 11d, 12a, and 16a2/b1 nodes were assigned to EI-4 nodes. The obligatory mediastinal LNM-1 nodes included only no. 110 station (12.4%), and the EI was 4.5. The rest of the No. 107~109 and No. 105~106 nodes were assigned to the EI-3A (1.1~1.5) and EI-4 categories (0.0~0.7), respectively.

### Obligatory station category of lymph node dissection for EGJ cancer

**Figure 5:**
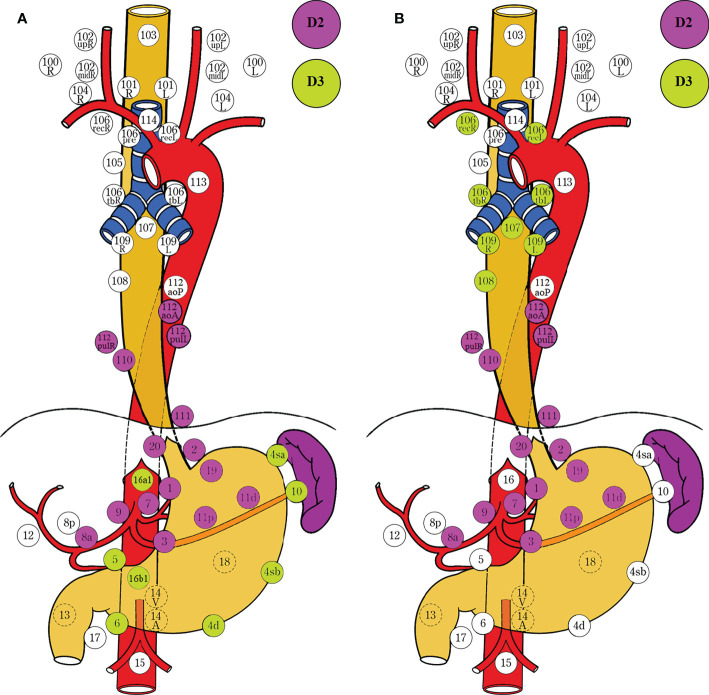
Obligatory and optional stations for lymph node dissection determined by the epicenter location and tumor extension in the stomach-predominant tumor **(A)** and esophagus-predominant tumor **(B)**. EI-1 and EI-2 nodes with EI exceeding 2% are designated as obligatory stations, while EI-3 nodes between 0.5% and 2% are designated as optional stations. The obligatory lymph nodes around the upper perigastric, lower mediastinal, and suprapancreatic zones should be resected in any case. The epicenter location has a greater impact on the metastasis at the obligatory stations. In contrast, tumor extension has a more important role on the metastasis at the optional stations. Lower perigastric and paraaortic nodes should be resected in the stomach-predominant tumor with gastric involvement exceeding 5.0 cm, and splenic hilar nodes in the Grecurvature involvement **(A)**. Upper and middle mediastinal nodes should be resected in the esophagus-predominant tumor with esophageal involvement exceeding 3.0 cm **(B)**. The D grading of nodal stations are marked with colored circles. D2 Complete dissection of EI-1 and EI-2 nodes. D3 Complete dissection of EI-1, EI-2, and EI-3 nodes. The left side (L) and the right side (R) should be distinguished for 101, 102, 104, 106rec, 106tb, 109, and 112pul. EI, efficiency index; LNM, lymph node metastasis.

Taking into account the benefit–risk balance, the obligatory stations with a lymph node metastatic rate exceeding 5% and an efficiency index over 2% should be resected in any case for D2 dissection. The nodal stations with highest efficiency and metastatic incidence from dissection were located at the pericardium and lesser curvature, suprapancreatic zone along at the root of the left gastric and the proximal splenic artery, and the lower esophagus, whereas the subsequent stations to be dissected were located at the celiac artery. The distal perigastric nodes were much less often metastatic and assigned to the EI-4 category. The efficiency index from middle mediastinal dissection showed marginal benefit from survival, and they were assigned to the EI-3A category. Superior mediastinal No. 105 and 106 nodes were assigned to the EI-3B/4 category. The metastasis to obligatory stations was largely dependent on the predominant epicenter location in terms the distribution of EI-1 and EI-2 nodes, and these obligatory zones for *en block* dissection as an “envelope-like” wrap were identified by the obligatory stations (**Figure 5A**).

### Optional station category of lymph node dissection for EGJ cancer

Overall, the optional stations with a lymph node metastatic rate of less than 5% and the efficiency index less than 2% were resected in selective cases, depending on the tumor extension. We did our best to search the published articles, but only the findings from prospective and large retrospective studies were adopted to identify the optional stations based on the predominant tumor location, tumor stage, tumor size, and involvement (**Table 4** **)**. The optional stations for dissection were largely determined by the location and grade of tumor extension as well as anatomic plane and *en block* dissection (**Figure 5B**).

**Table 4:** The optional stations for lymph node dissection based on the epicenter location and tumor extension.

Node zone	Multicenter study	Type	Risk factors for LNM	Category
**Perigastric**			**Stomach-predominant tumor**	
Nos. 1, 2, 3	Kurokawa et al. (36)	Prospective	Any case	LNM-1
Nos. 4d~6	Kurokawa et al. (36)	Prospective	Tumor size >6.0 cm	LNM-1
	Fujita et al. (19)	Retrospective	Stomach-predominant	EI-3A
			Esophagus-predominant	EI-3B
	Yamashita et al. (33)	Retrospective	Esophagus-predominant T3-4 ≤4.0cm	EI-3
			Stomach-predominant T3-4 ≤4.0cm	EI-3
	Lin et al. (37)	Retrospective	Tumor size >4.0 cm	EI-1
			Tumor size<4.0 cm	EI-3A
	Mine et al. (38)	Retrospective	Gastric involvement >5.0 cm	EI-1
**Suprapancreatic**			**Advanced EGJ cancer**	
Nos. 7, 8a, 9,11p	Kurokawa et al. (36)	Prospective	Any case	LNM-1/2
**Paraaortic**			**Gastric involvement**	
Nos. 16a2, b1	Motoori et al. (39)	Prospective	Gastric involvement >5.0 cm	LNM-1
			Tumor size >6.0 cm	LNM-1
			No. 2 or 7 nodes (+)	LNM-2
			Nos. 2 and 7 (+)	LNM-1
	Kurokawa et al. (36)	Prospective	Tumor size >6.0 cm	LNM-1
**Splenic hilar**			**Grecurvature-predominant**	
No. 10	Sano et al. (40)	Prospective	No	LNM-3
	Maezawa et al. (41)	Retrospective	Yes	LNM-2
	Yura et al. (42)	Retrospective	Yes	LNM-2
	Kano et al. (43)	Retrospective	Yes	LNM-1
**Mediastinal**			**Esophageal involvement**	
Nos. 110~112	Kurokawa et al. (36)	Prospective	>2.0 cm	LNM-2
Nos. 107~109			>3.0 cm	LNM-2
Nos.105~106			>3.0 cm	LNM-2
			**Esophagus-predominant**	
Nos. 110~112	Fujita et al. (19)	Retrospective	Yes	E-1/2/3A
Nos. 107~109			Yes	E-3A/2/3B
Nos. 105~106			Yes	E-3A/3B/4

The optional nodes zones were investigated by the risk factors including the predominant tumor location, tumor size, and length of gastric or esophageal involvement from the large-scale multicenter studies.

LNM, lymph node metastasis; EI, efficiency index; LNM-1, LNM ≥10%; EI-1, EI ≥5; LNM-2, LNM ≥5%; EI-2, EI ≥2; LNM-3, LNM<5%; EI-3, EI ≥0.5 (EI-3B ≥1.0% and EI-3B ≥0.5%). EI-1 and EI-2 nodes expressed as obligatory stations for dissection, while EI-3A and EI-3B as optional stations.

The lymph node metastasis to the lower perigastric stations was much less frequent for EGJ tumors smaller than 4.0 cm and delegated to the optional EI-3 category for dissection. In contrast, it can move to the obligatory EI-2 category in the stomach-predominant tumor larger than 4.0 cm and even to the EI-1 or LNM-1 category when the tumor size was bigger than 6.0 cm or gastric involvement exceeded 5.0 cm. The para-aortic lymph nodes (16a2/b1) were categorized as LNM-1 category when gastric involvement exceeded 5.0 cm or no. 2 or 7 node metastasis, especially for the less curvature-predominant tumor. Splenic hilar nodes were classified as LNM-2 category in the Grecurvature-predominant or Borrmann IV type tumor; only the LNM-3 category in the non-Grecurvature-predominant tumor. The mediastinal metastasis was frequent in the esophagus-predominant tumor, whereas it was rare in the stomach-predominant tumor. The lower mediastinal nodes were designated as LNM-2 category when esophageal involvement exceeded 1.0 cm, and the middle and upper mediastinal nodes as LNM-2 category when esophageal involvement exceeded 3.0 cm, respectively (**Table 4**).

## Discussion

The required extent of lymph node dissection for advanced EGJ adenocarcinoma was discordantly defined by the Japanese Classification of Esophageal Cancer (15) (**Figure 1A**) and Gastric Carcinoma Treatment Guideline (14) (**Figure 1B**), based on the incidence of lymph node metastasis from previous retrospective studies. Due to the lack of the estimated benefit from dissection at each station, we conducted an international survey on the extent of D2 dissection for EGJ carcinoma on the grounds of the therapeutic efficacy index (rate of metastasis × 5-year survival rate of patients with metastasis) derived from retrospective studies (18, 19). In the study, we investigated the nodal involvement and therapeutic efficacy from 16 retrospective studies which included 6,350 patients. According to the prespecified D2 dissection rule of LNM (%) exceeding 5% and EI over 2% in this study (18, 19, 36), the obligatory lymph nodes located at the lower mediastinal no. 110 station; upper perigastric no. 1, 2, and 3 stations; and suprapancreatic no. 7, 9, and 11p stations should be dissected in any case with advanced EGJ cancer (**Figure 4**). In contrast, the other abdominal and mediastinal stations, including the distal perigastric no. 4sb~6, splenic hilar no. 10, and para-aortic no. 16a2/b1 nodes as well as the middle and upper mediastinal nodes, needed not be routinely resected because of less metastasis and marginal benefits. Considering the benefit–risk balance, an optional station for *en block* dissection depended on the predominant location (epicenter location) and tumor extension (Borrmann type III and IV) (19, 33, 36, 38, 39, 44–46). The lower perigastric nodes should be resected in the stomach-predominant tumor with gastric involvement exceeding 5.0 cm, whereas the middle and upper mediastinal nodes should be resected in the esophagus-predominant tumor with esophageal involvement exceeding 3.0 cm, splenic hilar nodes in the Grecurvature-predominant tumor, and the paraaortic nodes in those with nodal involvement at the left pericardia or left gastric artery. Based on the findings, we deduced that the lower mediastinal, upper perigastric, and suprapancreatic nodes to be resected should be required for D2 dissections as an “envelope-like” wrap in any advanced EGJ cancer. In contrast, the optional stations for dissection depended on the area occupied by the tumor extension.

### The dissection grading of lymph node metastasis

The beneficial effect of lymph node dissection depends on *en block* dissection according to the metastatic grading at each station for EGJ carcinomas, especially for complete dissection of group 1 and group 2 nodes as the standard D2 dissection for gastric cancer (14, 15). In curable cases of advanced gastric cancer, D2 dissection (nodal dissection up to group 2 nodes) is associated with better prognosis than D1 dissection (dissection of perigastric nodes only). The D grading (the extent of lymph node dissection) is required to be larger than the anatomic N grading (the spread of lymph node metastasis) for complete R0 resection when the metastasis to adjacent lymph nodes is limited to the ability but for an excessive dissection to the inability of the surgeon to excise widely all tissues (15, 17). Thus, the N grading depends on not only the number of positive nodes (N-stage) and the sites (stations) of lymph node metastases but also qualitatively according to the therapeutic benefit (regional nodes) (18). Ideally, the most accurate surgical dissection for nodal metastasis is D equal to N rather than D greater than N. However, the preoperative detection of lymph node metastasis is difficult, let alone identify the grading of dissection. The therapeutic efficacy index of estimated survival benefit from lymph node dissection, calculated by multiplying the metastatic incidence to each station by the corresponding 5-year survival rate, is a more useful method to express the extent of lymph node metastasis as a D grading (18, 19), irrespective of any other tumor stage. Thus, the efficiency index takes into account not just the distribution and incidence of metastatic nodes but also the estimated survival benefit from each metastatic station (18). The order of efficiency index was consistent with that of the corresponding LNM rate from each station. Accordingly, LNM-1 and LNM-2 nodes with rates exceeding 5% correspond exactly to the EI-1 and EI-2 nodes of EI exceeding 2%, whereas LNM-3 nodes with rates less than 5% correspond to EI-3 and EI-4 nodes of EI less than 2% in our study. Considering *en block* dissection and benefit–risk balance, LNM-1/2 and EI-1/2 nodes were designated as obligatory lymph node stations to be dissected in any EGJ carcinomas, whereas LNM-3 and EI-3 nodes designated as optional stations need not be dissected routinely unless in a low risk for morbidity and mortality. The EI-4 nodes as distant metastasis need not be dissected in any case. Although many dissenters argued that it was not the best way to determine the extent of lymph node dissection based on the metastatic distribution, there was not an accurate method to replace it, since the regularity of lymphatic flow is very abundant and complex from the EGJ zone between stomach and esophagus (47, 48). Currently, the D grading of lymph nodes is more favorable than the N grading for identifying the obligatory stations with the highest metastatic rate and greatest benefit from dissection, and the optional stations with much less frequent metastasis and marginal benefit, and also avoiding unbeneficial dissection for those with an extensive involvement (**Table 1**).

### Obligatory lymph node D2 dissection determined by epicenter location

Overall, large retrospective or prospective studies have reached consensus on the obligatory stations for dissection at the upper perigastric, suprapancreatic, and lower mediastinal zones based on nodal metastasis from each station (8–11, 19, 21, 33, 36, 49) (**Figure 5A**). There is a general rule from the guidelines that the incidence of nodal metastasis is positively related to the pathological T stage that determines the prognosis, whereas the lymph node metastasis distribution is strongly associated with the tumor location that determines the range of surgical dissection. The former is quantitatively expressed by N stage, whereas the latter is locally expressed by nodal stations. Obviously, the metastatic stations to be dissected depend on the classification of tumor location, including the epicenter and the tumor extension, because lymph node metastasis spreads from the tumor location, *via* the lymph vessel along the artery, and to the lymph node station. EGJ cancer shares the bidirectional pathway of the lymphatic reflux from the lower esophagus and the upper stomach at the EGJ zone. It begins with the upper perigastric and lower paraesophageal nodes (group 1), which pass *via* the suprapancreatic nodes along the celiac artery and the lower mediastinal nodes along the left subphrenic artery (group 2), to the para-aortic nodes (group 3). Accordingly, the lymph node metastases to the obligatory stations for dissection were largely dependent on the epicenter location defined by Nishi’s and Siewert’s classification as a relatively constant zone (**Figures 2A, B**). The findings clearly displayed that the obligatory nodes for dissection were located at the lower mediastinal, upper perigastric, and suprapancreatic zones.

However, the nodal metastasis of optional stations may depend more on the tumor extension rather than the predominant tumor location, and then the grading of tumor extension is not well described by both of the classifications. Thus, it remains debatable on the optional dissection at the distal perigastric, para-aortic, splenic hilar, and middle as well as superior mediastinal stations. The esophagus-predominant tumors are more prone to mediastinal metastasis depending on the extent of esophageal invasion, whereas the stomach-predominant tumors are more likely to develop abdominal metastasis depending on the extent of gastric involvement toward the different gastric region in terms of the lymphatic flow routes from the marked geographic distributions of epicenter location and tumor extension. We failed to reveal the indications for optional stations dissecting due to the limitations of the material. Therefore, these stations were discussed in detail, based on the prospective or large retrospective studies.

### Optional dissection of distal perigastric lymph nodes

The extended gastrectomy with inferior esophagectomy was performed in most of advanced cases with the stomach-predominant tumor, because nodal metastasis was frequently involved in the abdominal stations by the tumor extension toward the gastric side (19, 21, 25, 33, 36, 50). In the findings, the highest incidence and efficiency index of abdominal stations to be dissected for EGJ cancer were the no. 1, 2, 3, 7, 9, and 11p nodes (EI-1 and EI-2 nodes) at the proximal perigastric and suprapancreatic stations except for the distal perigastric no. 4sa, 4sb, 4d,5, and 6 nodes (EI-3 nodes). It was consistent with the low incidence of metastasis and marginal benefit from dissection at the distal perigastric stations (No. 4sa~6 nodes) (19, 29, 30, 33, 36). According to the EI and LNM categories for a stomach-predominant tumor, EI-1 (LNM-1/N1) and EI-2 (LNM-2/N2) nodes can be resected by transhiatal proximal gastrectomy with partial esophagectomy. In order to resect EI-3 nodes, the supporters argued that the distal perigastric nodes should be resected for survival benefits, especially in the stomach-predominant tumor. Thus, the extended gastrectomy remains a prevalent procedure for EGJ cancer in Asia, irrespective of gastric involvement (14). The predominant tumor location, stage, and size were considered as the important indications for extended gastrectomy (37, 38). However, a multicenter study (19) reviewing 1,289 patients with the EGJ tumors indicated that the no. 4d~6 nodes should be assigned to EI-3 and need not be dissected because of low metastatic rate and poor prognosis, even in the stomach-predominant tumor. Although the large-sample multicenter study revealed that proximal perigastric, suprapancreatic, and inferior mediastinal dissections were the most essential zones for advanced EGJ cancer, a further analysis on the extent of gastric involvement was lacking. Similarly, the Japanese Gastric Association and Esophageal Society Joint Working Group proposed the optimal extent of lymphadenectomy for 2,807 patients with EGJ cancer of less than 4.0 cm in diameter (33). They determined that the metastatic rate was very high in the no. 1, 2, 3, and 7 nodes; moderate in the no. 8a, 9, 11p, and 110 nodes; and low in the no. 4sa, 4sb, 4d, 5, and 6 nodes. Even in the stomach-predominant cancer at stages T3–T4, the rates of no. 4sa and 4sb were 1.0% and 0.8%, respectively. Accordingly, the distal perigastric node dissection seemed to be unable to offer survival benefits. The findings confirmed very low metastatic rates (0.3%~2.1%) and an efficiency index (0%~0.5%), suggesting that the dissection along the lower perigastric portion was unnecessary unless in the case of tumors larger than 4.0 cm in terms of *en block* dissection, and also the potential benefit was observed only in a retrospective study (37). Another multicenter study of 288 patients with advanced Siewert type II carcinoma stated that the extent of metastasis to the distal perigastric nodes was associated with the distance from the EGJ to the distal end of gastric involvement (38). When the distance ≤30 mm, the metastasis rate of at least one lymph node was 2.2%; proximal gastrectomy is sufficient. In contrast, when the distance ≥50 mm, the rate was 20.0%; a total gastrectomy should be considered. The distance was 30~50 mm, a rate of 8.0%; the choice should satisfy the surgical requirements. The prospective multicenter study conducted by Kurokawa et al. (36) unequivocally displayed that LNM-1 nodes included abdominal stations 1, 2, 3, 7, 9, and 11p, whereas LNM-2 nodes included abdominal stations 8a and 19 and lower mediastinal station 110 only. Subgroup analysis based on the tumor size showed that the metastasis rate of at least one of perigastric stations 4d, 5, or 6 reached 10.7% in cases with a tumor size bigger than 6.0 cm. However, the survival benefit remains waiting.

These findings described above seem to imply that the tumor extension associated with tumor size (gastric involvement) had a greater impact on the distal perigastric lymph node metastasis than the predominant tumor location and T stage. Since both Siewert’s and Nishi’s classification clearly settled the epicenter location within 2.0 cm above and below the EGJ, irrespective of tumor size in the former and histological type in the latter, and proximal and distal involvement as well as macroscopic classification in both (15). Considering the immovable epicenter location, the distal perigastric metastasis might depend more on the extent of tumor extension (macroscopic types) rather than on the epicenter location (deepest tumor invasion). Moreover, the predominant tumor location and size might affect the extent and direction of gastric involvement associated with the metastatic pattern of lymph nodes. Unfortunately, the majority of included studies located the epicenter 2.0 cm above and below the EGJ alone according to Siewert’s and Nishi’s classification but lost the subgroup analysis on the tumor extension into different gastric regions. Only by a logical analysis in this way was it reasonable to explain the similar low LNM rate and EI index in both stomach-predominant and esophagus-predominant tumors, but they were sharply increased in gastric involvement by more than 5.0 cm or tumor size by over 4.0 cm. In clinical practice, the type of gastrectomy limits the extent of lymph node dissection, and also the metastatic distribution affects the type of gastrectomy. In fact, extended gastrectomy was widely performed in EGJ cancer because of adequate distal margin and reflux symptoms caused by proximal gastrectomy rather than *en block* dissection. The survey aimed at the extent of nodal dissection larger than the spread of nodal metastasis for complete R0 resection but rather the gastrectomy. Based on the discussions from the findings above, the proximal gastrectomy with lower esophagectomy should be an optimal procedure in the stomach-predominant tumor small than 4.0 cm, whereas extended gastrectomy should be considered in those of gastric involvement more than 5.0 cm in terms of *en block* dissection and sufficient distal margins. In other words, the para-pyloric (no.5 and 6 stations) dissection may be required for an en block dissection when the EGJ tumors are large enough to involve the right gastric artery and the right gastroepiploic artery ( Borrmann III or IV type). A prospective trial on tumor location and extension remains highly warranted for this population.

### Optional dissection of paraaortic lymph nodes

Paraaortic lymph node dissection is not a standard treatment for EGJ cancer (14, 15). However, several studies have reported a high incidence of metastasis and the most frequent site of recurrence to para-aortic stations among patients with EGJ cancer (22, 33, 34, 51). Moreover, the finding showed that para-aortic no. 16a2/b1 nodes with a rate of 2.5% (0%~14.4%) less than 5% and EI (0.0~1.9) less than 2.0 were classified as LNM-3 and EI-4 nodes. The finding was obviously inconsistent with that of LNM-1 (17%, 14.4%, and 22%) and EI-2 (3.2, 2.4, and 4.9) nodes in retrospective studies reported by Mine et al. (22), Yoshikawa et al. (34), and Nunobe et al. (51), respectively. However, it was consistent with the overall rate of 4.7% and was thus classified as LNM-3 with a rate less than 5% in the prospective multicenter study reported by Kurokawa et al. (36). Subgroup analysis showed that the rate of no. 16a2 was 10.1% if the tumor size exceeded 6.0 cm, but therapeutic efficiency remains ongoing for availability. These retrospective findings were conflicted with those from the prospective study but completely consistent with those of tumor sizes larger than 6.0 cm, which indicated a selective bias from the small retrospective study. Therefore, we combined the latest findings to determine the necessity and effectiveness of paraaortic dissection based on the efficacy index. Unexpectedly, the finding consistent with that of the prospective one consolidated that no. 16a2/b1 nodes should not be routinely resected as LNM-3 nodes. Due to the lack of the material, we failed to perform a subgroup analysis based on tumor size, tumor stage, and the correlation among metastatic stations to identify those subsets with a high therapeutic efficacy. However, Motoori et al. (39) identified a metastatic rate of 4.7% among 344 patients in the prospective multicenter study on the paraaortic dissection in advanced EGJ cancer. The independent risk factors for no. 16a2 metastasis were at stage N2-3 (11.8%), metastasis at the no. 2 and 7 nodes (23.7%), gastric involvement exceeding 5.0 cm (12.5%), and tumor size bigger than 6.0 cm (10.1%). This revealed that the lymph nodes from EGJ zone can easily metastasize to terminal stations, para-aortic nodes, along the left gastric artery (no.7 nodes) and the subphrenic artery (no.2 nodes) as long as the EGJ tumor infiltration is sufficient to involve the mesogastric vessels. Based on the findings combined with those of the prospective trial, we inferred that para-aortic lymph nodes need not to be resected routinely unless in the stomach-predominant cases with positive nodes at the no. 2 or 7 station or gastric involvement exceeding 5.0 cm. As an extensive involvement, paraaortic dissection should be performed cautiously based on the epicenter location and gastric involvement in terms of *en block* dissection. The further prospective trials on the no. 16a2/b1 metastasis after neoadjuvant chemotherapy are highly warranted for beneficial effects.

### Optional dissection of splenic hilar lymph nodes

The splenic hilar node dissection is not a standard treatment for EGJ cancer (14). The lymphatic flow from no. 4sa and 4sb stations along the greater curvature can metastasize to splenic hilar nodes (no. 10) (48). Therefore, Grecurvature-predominant tumor involvement is an independent risk factor for lymph node metastasis in the splenic hilum (41–43). In fact, the less curvature-predominant tumor is still the most common type for EGJ cancer. Regularly, the findings showed a low incidence of only 3.4% at the splenic hilar nodes, and EI was 0.8% as EI-3B. Meanwhile, randomized trials (40) showed that the splenectomy in the upper gastric cancer of no Grecurvature involvement could not improve the prognosis compared with spleen preservation. This result may be applicable for EGJ tumors. The dissection at the splenic hilum should be optional rather than obligatory. Even in the Grecurvature-predominant tumor, the benefit from splenic hilar dissection is lacking.

### Optional dissection of mediastinal lymph nodes

Regarding mediastinal nodes, the incidence of metastasis was quite high for patients with EGJ cancer in previous studies (51–54). The findings showed that the highest incidence and efficacy of mediastinal nodes to be dissected was only no. 110 nodes with a metastatic rate of 12.4%, whereas the rest of the no. 105~112 nodes were assigned to LNM-3 (0%~2%) and EI-3 or EI-4 (0.0~1.5), respectively. Middle mediastinal nodes at the no. 107~109 stations (1.1~1.5) were assigned to the EI-3A category, superior mediastinal nodes at the no. 105~106 stations (0.0~0.7) to EI-4. According to the EI category, EI-1 and EI-2 nodes can be resected by transhiatal lower esophagectomy with proximal gastrectomy. Middle and upper mediastinal dissection was not routinely required in case of limited esophageal invasion less than 3.0 cm. Particularly, the JCOG9502 trial (8, 9) showed that left transthoracic mediastinal dissection provided no advantage over transhiatal lower mediastinal dissection for patients with EGJ cancer with esophageal invasion less than 3.0 cm. A multicenter retrospective study (55) showed that the metastatic rate of the upper or middle mediastinal nodes was higher when esophageal involvement exceeded 3.0 cm, whereas the rate was higher in the lower mediastinal nodes when involvement exceeded 2.0 cm. One of the most popular explanations was that the potential metastasis was correlated with the length of esophageal involvement (36, 45, 46, 55). Ultimately, Kurokawa et al. (36) conducted the first prospective multicenter to confirm that the overall rate of the mediastinal no. 110 nodes was 9.3%, and the rate of at least one of the lower mediastinal nodes was 13.3%. Subgroup analysis showed that the metastasis rates at the no. 111 and 112 stations were less than 5% unless esophageal involvement exceeded 4.0 cm (10.7% and 7.1%). In contrast, the rate at the no. 110 station exceeded 10.8% if esophageal involvement exceeded 2.0 cm. It determined that dissecting no. 110 nodes were sufficient unless esophageal involvement exceeded 4.0 cm, and dissecting no. 110 was unnecessary unless esophageal involvement exceeded 2.0 cm. The incidence of at least one of superior mediastinal nodes was 6.1%, and that of the middle mediastinal nodes was 7.1% if esophageal involvement exceeded 3.0 cm. Accordingly, in order to resect the EI-3 (LNM-3) nodes, the subtotal esophagectomy with proximal gastrectomy is required in an esophagus-predominant tumor with esophageal involvement exceeding 3.0 cm. Nevertheless, the therapeutic efficacy of dissection from each of the involved stations remains to be unavailable. Lacking the materials, we failed to perform the analysis based on the esophageal involvement, which is an independent risk factor of mediastinal metastasis. However, the survival benefits from upper and middle mediastinal dissections were questionable in Siewert II tumor (8, 9, 56) and even in Siewert I tumor (10, 11) from RCT studies. It was worthwhile for adequate margin and *en block* dissection (36). Based on the prospective findings, the lower mediastinal dissection was obligatory in any case with EGJ cancer, whereas upper and middle mediastinal dissection was optional for the esophagus-predominant tumor with the esophageal involvement exceeding 3.0 cm. Because the lymph nodes around the lower esophagus are mainly metastasized along the left gastric artery and the subphrenic artery downward to the para-celiac stations, while those nodes from the middle and upper segment are mainly metastasized along the bronchial artery, the recurrent laryngeal nerve and the lower thyroid artery upward to the middle and upper mediastinal stations. It can be explained that the range of tumor extension has precedence over the epicenter location in the middle and upper mediastinal metastasis for an esophagus-predominant tumor (47, 48).

### Comprehensive standpoints and critical comments

Overall, the obligatory stations for dissection are largely determined by the epicenter location, whereas the optional stations depend on the tumor extension. The epicenter indicates the vertical depth of deepest tumor invasion related to the tumor size, whereas the tumor extension represents the horizontal length of tumor invasion associated with the macroscopic types. The current classification of gastric cancer and esophageal cancer regards the epicenter location to be the most important factor for defining regional lymph node grouping, irrespective of macroscopic classification. Furthermore, even in infiltrative ulcerative and diffuse infiltrative tumors, the epicenter location still has precedence over the tumor extension in defining regional lymph node grouping. According to the guidelines, both mass and infiltrative cancers are considered to have the same lymph node grouping in the epicenters located in the same anatomic region. In fact, both epicenter location and tumor extension have greater effects on determining the regional lymph node grouping in terms of the lymphatic drainage. In addition, even in infiltrative carcinomas, the tumor extension should have precedence over the epicenter location in determining the regional lymph node grouping because of a wide extension. Thus, both of them should be considered in practice. However, both Siewert’s and Nishi’s classifications only defined the epicenter location, irrespective of tumor size in the former, histological type in the latter, and tumor extension as well as macroscopic type in both (**Figure 2A, B**). Accordingly, a unified classification for EGJ cancer had to be developed for improving the surgical quality based on the epicenter location with tumor extension, especially for esophageal and gastric involvement. On the one hand, the epicenter location should be described as the esophageal side (E) and gastric side (G), and the distance between the epicenter and the EGJ is recorded (7). On the other hand, tumor extension into the esophagus or stomach should be described by the distance from the EGJ to the proximal or distal end of the tumor, whereas the extending location into the stomach is described as lesser (Less) and greater (Gre) curvatures, and the anterior (Ant) and posterior (Post) walls, and circumferential involvement (Circ) based on the four equal parts of gastric circumference (17). It is beneficial to eliminate the heterogeneity from the epicenter location (depth of tumor invasion) and tumor extension (macroscopic types). EGJ cancer should be defined as adenocarcinoma with an epicenter located within 2.0 cm of the EGJ and scheduled as three subtypes as the following: E type, epicenter location at the oral side of the EGJ and esophageal involvement exceeding 3.0 cm; E-G type, epicenter location between the oral and anal sides of the EGJ, esophageal, and gastric involvement within 3.0 and 5.0 cm respectively; and G type, epicenter location at the anal side of the EGJ with gastric involvement exceeding 5.0 cm.

In a few words, the priority and optimal extent of D2 lymph node dissection of EGJ cancer remain focused on those nodal stations with frequent metastasis. These positive stations actually represent a region of lymphatic reflux, so those positive stations along with adjacent ones should be dissected along the anatomical plane as an “envelope-like” wrap. Further on, the therapeutic efficacy from *en block* dissection at nodal stations with higher positive rates should be clarified as the survival benefit. Therefore, the lymph node dissection for EGJ cancer should consider at least the following important factors: epicenter location, tumor extension, Borrmann type, and tumor T stage as well as anatomical plane and *en block* dissection. Based on the comprehensive findings assessing the metastatic pattern and therapeutic efficacy in the large-scale international survey of 6,350 patients, we determined that the obligatory nodes with highest metastatic incidence and therapeutic efficiency located at the no. 1, 2, 3, 7, 9, 11p, and 110 stations and should be resected in any case. The no. 5, 8a, 12a, 11d, 19, and 20 stations should be considered for an “envelope-like” wrap dissection, whereas the optional nodes for dissection depended on the tumor extension. Middle and superior mediastinal nodes should be resected in the esophagus-predominant tumor with esophageal invasion exceeding 3.0 cm, whereas distal perigastric and paraaortic dissections should be resected in the stomach-predominant tumor with gastric involvement over 5.0 cm, and splenic hilar dissection in the Grecurvature-predominant tumor or Grecurvature involvement considering *en block* dissection. In practice, the surgical approach is governed largely by the dissection D larger than metastatic N grading (14, 15, 17, 20). On the one hand, for a stomach-predominant tumor, the obligatory stations with EI-1 and EI-2 nodes can be resected by proximal gastrectomy with lower esophagectomy *via* a transhiatal approach, whereas extended gastrectomy is required for the distal resection margin and excessive dissections at the distal perigastric and paraaortic stations with optional EI-3 nodes when gastric involvement exceeded the upper stomach or Borrmann IV type (**Figure 5A**). On the other hand, for an esophagus-predominant tumor, the obligatory nodes can be resected by the lower esophagectomy with proximal gastrectomy *via* a transhiatal approach, whereas the subtotal esophagectomy with proximal gastrectomy *via* a transthoracic approach is required for complete mediastinal dissection and adequate margin when esophageal involvement exceeded 3.0 cm (**Figure 5B**). The survival benefits from dissections at optional stations remain worthy of prospective evaluation on the paraaortic and mediastinal dissection based on the grade of tumor extension (57).

### Limitations of the study

This study has some limitations. Firstly, the included studies are retrospective; secondly, some mediastinal stations are simply merged for *en block* dissection; thirdly, we failed to determine the optional stations of lymph node metastasis and adopted the findings from the three prospective and large multicenter studies based subgroup analysis on the tumor extension.

## Conclusions

The obligatory D2 dissection stations for patients with locally advanced EGJ cancer included the lymph nodes around the pericardia and lesser curvature; left gastric artery, celiac artery, and proximal splenic artery; and lower mediastinum. In contrast, other optional lymph node stations for dissection should be indicated by the predominant tumor location and tumor extension. The obligatory stations can be resected by the proximal gastrectomy with lower esophagectomy *via* a transhiatal approach, whereas optional station dissection was indicated by the degree of tumor extension into gastric regions. The extended total gastrectomy is required for lower perigastric dissection in the stomach-predominant tumor with gastric involvement exceeding 5.0 cm, whereas subtotal esophagectomy *via* a right thoracic approach is required for complete mediastinal dissection in the esophagus-predominant tumor with esophageal involvement exceeding 3.0 cm.

In conclusion, we deduced that the upper perigastric, suprapancreatic, and lower mediastinal nodes should be routinely resected as an “envelope-like” wrap in any case for a mandatory D2 dissection standard.

## Author contributions

BZ and RL make substantial contributions to conception, design, creating digital artwork, and acquisition, analysis and interpretation of data. BZ drafted the paper. RW and XB revised it critically for crucial intellectual content. All authors contributed to the article and approved the submitted version.

## Funding

The Shanxi Provincial Science and Technology Key Project (no. 20140313011-3) supported this work.

## Conflict of interest

The authors declare that the research was conducted in the absence of any commercial or financial relationships that could be construed as a potential conflict of interest.

## Publisher’s note

All claims expressed in this article are solely those of the authors and do not necessarily represent those of their affiliated organizations, or those of the publisher, the editors and the reviewers. Any product that may be evaluated in this article, or claim that may be made by its manufacturer, is not guaranteed or endorsed by the publisher.
